# Arts-Based Interventions for Professionals in Caring Roles During and After Crisis: A Systematic Review of the Literature

**DOI:** 10.3389/fpsyg.2020.589744

**Published:** 2020-12-22

**Authors:** Dominik Havsteen-Franklin, Megan Tjasink, Jacqueline Winter Kottler, Claire Grant, Veena Kumari

**Affiliations:** ^1^CNWL NHS Foundation Trust, London, United Kingdom; ^2^Brunel University London, Uxbridge, United Kingdom; ^3^Barts Health NHS Trust, London, United Kingdom; ^4^Royal Free London NHS Foundation Trust, London, United Kingdom

**Keywords:** care professionals, arts therapies, stress, trauma, crisis, pandemic, systematic review

## Abstract

Crisis events, such as the COVID-19 pandemic, can have a devastating effect on communities and the care professionals within them. Over recent years, arts-based interventions have helped in a wide range of crisis situations, being recommended to support the workforce during and after complex crisis but there has been no systematic review of the role of arts-based crisis interventions and whether there are cogent themes regarding practice elements and outcomes. We, therefore, conducted a systematic review to (i) define the arts-based change process used during and after crisis events, and (ii) explore the perceptions of intermediate and long-term mental health benefits of arts-based interventions for professionals in caring roles. Our search yielded six studies (all qualitative). All data were thematically aggregated and meta-synthesized, revealing seven practice elements (a safe place, focusing on strengths and protective factors, developing psychosocial competencies to support peers, emotional expression and processing, identifying and naming the impact of the crisis, using an integrative creative approach, and cultural and organizational sensitivity) applied across all six studies, as well as a range of intermediate and long-term benefits shared common features (adapting, growing, and recovering; using the community as a healing resource; reducing or preventing symptoms of stress or trauma reactions, psychophysiological homeostasis). The ways in which these studies were designed independently from one another and yet used the same practice elements in their crisis interventions indicates that there is comparability about how and why the arts-based practice elements are being used and to what effect. Our findings provide a sound basis and meaningful parameters for future research incorporating quantitative and qualitative approaches to firmly establish the effectiveness of art-based interventions, and how arts can support cultural sensitivity, acceptability and indicated outcomes, particularly those relating to stress and trauma during or following a crisis.

## Introduction

The recent COVID-19 pandemic resulted in care professionals being thrown into pressurized working environments, adapting their skills and demonstrating professional responsiveness and flexibility. The pandemic reached a critical peak that stretched physical, community, social and care resources. Crises take many forms and have a significant impact on populations. Recent research suggests that there are comparable social changes between a range of crises, for example COVID-19 and natural disasters (Shelby and Tredinnick, 1995; Miles and Shipway, 2020; Mostafanezhad, 2020). After examining substantial research, Perry (2018) also resolves that crises are defined by the level and nature of human interactions relating to the event. This is evidenced by severe social reactions, for example, disillusionment (Centers for Disease Control Prevention, 2005), depression (Yildirim et al., 2020), and fear exacerbated by media coverage (Garfin et al., 2020). The impact of crises on the mental health of care professionals is varied (Gavin et al., 2020; Zhang et al., 2020) however first impressions suggest a rise in depression (Rajkumar, 2020), stress, paranoia, fear, traumatic responses, and bereavement complications (Chaturvedi, 2020; Ho et al., 2020; Huang et al., 2020; Rajkumar, 2020; Torales et al., 2020). As, according to Perry (2018) and the available evidence, a crisis is described by human interactions, we conclude that interventions to help care professionals with their mental health and roles need to relate to the relational context and therefore are required to be accessible and aligned with the professional needs of carers. Therefore, according to recent research, a successful crisis intervention is required not only to be helpful for individual care professionals, but should also focus on how to enable successful community recovery (Rubin, 1985; Stehr, 2001; Chang and Miles, 2003; Jimerson et al., 2005; Kulig et al., 2014).

Arts therapies have been widely used in mental health services to treat a range of mental health issues (Karkou and Sanderson, 2006; Dunphy et al., 2013; Zubala and Karkou, 2018). Over recent years the arts therapies have been offered alongside other treatments to help in a broad range of crisis situations, being recommended to support the workforce during and after complex emergencies (Surya et al., 2017). Given the multifarious nature of the crisis and the impact on health and well-being, we aim to systematically investigate the role of arts-based crisis interventions and whether there are cogent themes regarding practice elements and perceived benefits. To date, there has not been a systematic review of literature in this area describing what an arts-based crisis intervention may look like and the perceived long-term benefits to the participants. This review reports and appraises findings from descriptive and qualitative studies regarding how arts-based practice is employed to support carers during and after a crisis.

## Methods

We conducted the literature search for the review from 12 to 18th May 2020 following PRISMA criteria (Moher et al., 2009). We initially conducted a Google Scholar search to determine the breadth of papers available. Following this, we conducted searches in CINAHL, Medline, PUBMED, SOCI INDEX, and PSYCH Info. The abstracts were searched using the search terms adjusted according to the database:

(“Health personnel” OR “Healthcare work^*^” OR Nurse OR Doctor OR “Hospital staff” OR “Health work^*^” OR “Care assistant” OR Pharmacist OR Physician) AND (“Mental illness” OR “Vicarious Trauma” OR “Emotional well-being” OR “Mental ill-health” OR Anxiety OR Depression OR “Self-harm” OR “Self-esteem” OR Confidence OR “Mental health” OR Suicid^*^ OR Loneliness OR Distress OR Aggression OR Stress) AND (Resilience OR “Subjective well-being” OR “Cognitive flexibility” OR “Post-traumatic growth” OR “Stress-related growth” OR Hardiness OR Optimism OR Hope) AND (“Art intervention” OR “Music Therap^*^” OR Dramatherap^*^ OR “Art Therap^*^” OR “Dance Movement Therap^*^” OR “Body Movement Therap^*^” OR “Art Psychotherap^*^ OR Psychodrama OR “Arts-Based” OR “Creative art^*^”)

**Figure 1:**
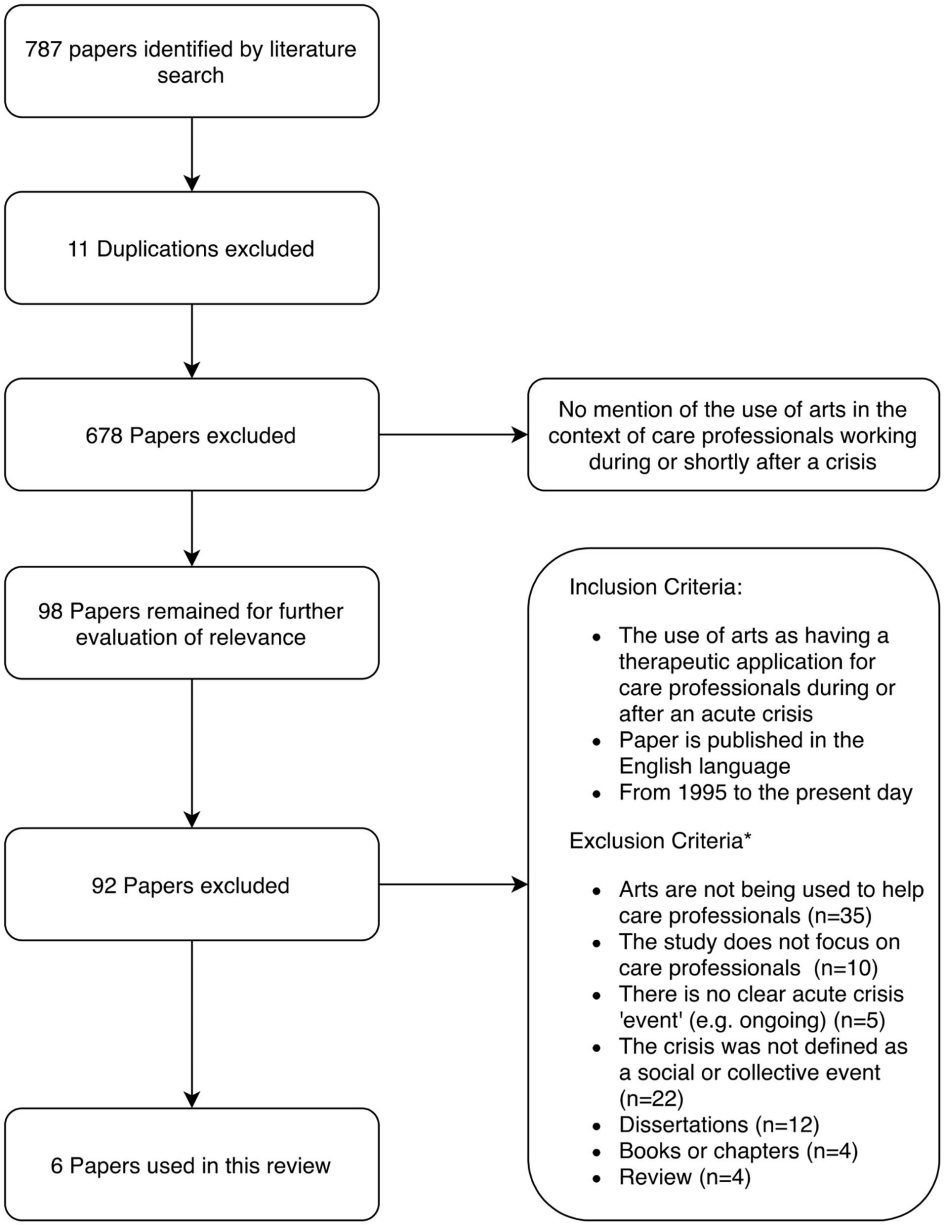
Search strategy based on the PRISMA flow diagram.

The references of relevant literature returned were reviewed and search terms compiled until it was felt that the search encompassed all relevant peer reviewed literature (Figure 1).

**Figure 2:**
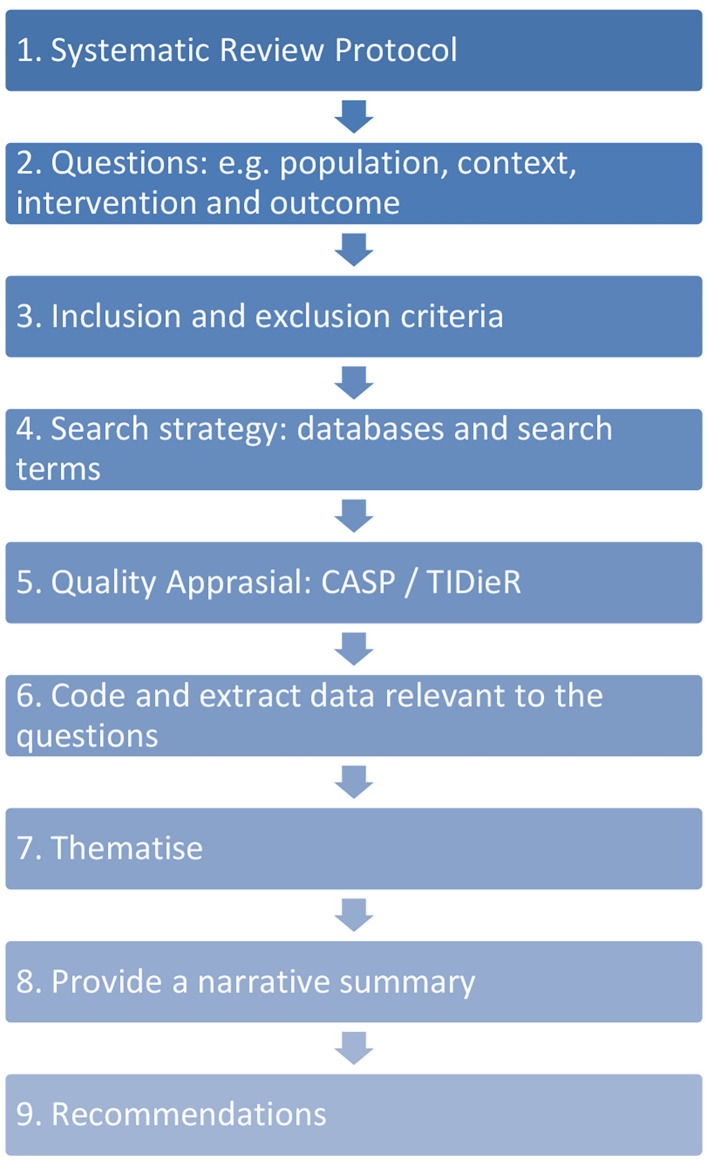
Systematic review process.

The initial search conducted in Google Scholar and 5 databases produced 787 papers that were considered relevant using the search terms. On reading the abstracts we excluded 689 papers because of the contexts within which the arts were being applied. We refined inclusion and exclusion criteria to focus on the question. We considered 98 papers through reading the abstracts or where the abstract was not sufficiently explicit about the inclusion criteria; we read the full text and determined that 92 of these did not meet our inclusion/exclusion criteria. The review team read the papers and shared findings, which led to further eliminations of papers to produce six papers for the review. There were no quantitative studies investigating effectiveness of the interventions. Therefore, a qualitative approach to reviewing the literature was considered appropriate and meets the review aim of making recommendations based on perceived benefits to using arts in response to a crisis to help care workers. We used a stepped model as described by Pearson (2004) (see Figure 2).

We conducted a thematic meta-synthesis of the selected papers. The review team of five people; four experienced arts therapists, and one psychologist, three of which had previously conducted systematic reviews, used Dedoose software to code the papers according to the questions. We revised the questions according to the review process and preliminary findings (see Table 1). We then coded excerpts that related to the following questions:

1. What are the change process elements of the arts-based intervention?
2. What are the perceived intermediate and long-term benefits of arts-based interventions?

**Table 1:** CASP Rating Criteria.

Was there a clear statement of the aims of the research?
Is the qualitative methodology appropriate?
Was the research design appropriate to address the aims of the research?
Was the recruitment strategy appropriate to the aims of the research?
Was the data collected in a way that addressed the research issue?
Has the relationship between researcher and participants been adequately considered?
Have ethical considerations been taken into consideration?
Was the data analysis sufficiently rigorous?
Is there a clear statement of findings?
How valuable is the research?

The question terms were agreed by the group and further refined as the review progressed. The context of an “acute crisis” was defined as an event that affected the community, where this was an unexpected occurrence, had a clear onset and affected how the community usually related and interacted. As the review progressed, the defined model of crisis that met our definition included pandemics (COVID-19), manmade disasters (e.g., buildings collapsing because of overbearing loads), community eviction, critical incidents, complex emergencies, genocide, national tragedy, natural disasters and human conflict (e.g., war). The definition of the carer included anybody in a professional role that was required to provide support and care for others subject to the direct impact of the crisis. These roles went beyond frontline health care workers and included social workers, community care workers, relief workers, nurses, key workers, medical students, emergency personnel, schoolteachers, training personnel, and community leaders.

### Coding

We decided the relevance of the papers on key content, including the type of crisis, the caring role, the arts applied, the name of the crisis model, group or individual format and the main health issue being addressed (See **Table 3**). These codes were then meta-synthesized, and the papers were read several times to determine the shared crisis model elements. The knowledge, skills and processing used to facilitate the interventions were interactional and the immediate impact was not possible to differentiate from the intervention description. Therefore, the themes reflected a collaborative process where cause and effect were less differentiated and that we named “change process elements.” For example, the facilitator establishing a safe place also enabled the participant to establish a safe place. This included the therapist's action within the intervention and the immediate impact.

Initially, a shortlisted paper was coded by the first author and examined by two co-authors. A second paper was then coded independently by three authors to determine any significant variation across coding and the use of the Dedoose software. Coding principles were agreed and included abbreviated, summarized and key words that could be used to categorize an excerpt. Any variation in coding was discussed, and we approved new codes and coding application. Once all six papers were coded, we discussed all papers and decided codes to prepare for meta-synthesizing the data into themes.

The codes were further analyzed for prevalence across the papers according to frequency and number of papers within which the themes appeared, to determine whether the themes formed a principal focus or were only coded in a minority of the papers. Coding and drawing themes from the papers was recorded at every step, using both Dedoose software and excel spreadsheets to summarize the findings for exploration by the authors. Criteria for papers to meet the review criteria were collated (See Table 1). The initial application of codes resulted in 480 codes applied 1,007 times across 567 excerpts. We grouped the codes according to superordinate codes that covered our two areas of inquiry; change process elements of the arts-based crisis model, and the perceived intermediate and long-term benefits of the crisis model.

### Quality Assessment

A fourth co-author independently rated the short-listed papers for quality using the Atkins' approach to critical appraisal (CASP) (Singh, 2013). Each paper was given a score out of 10 based on 10 questions (Table 2).

**Table 2:** CASP ratings.

References	Q1	Q2	Q3	Q4	Q5	Q6	Q7	Q8	Q9	Q10	Rating
Herbert (2018)	1	1	1	0	0.5	0.5	0.5	0	0.5	1	6
Ho et al. (2014)	1	0.5	0	0.5	0.5	0.5	1	0	1	1	6
Huss et al. (2010)	1	1	1	1	1	0	1	1	1	1	9
Potash et al. (2020)	1	0.5	0	0	0	0	0	0	0.5	1	3
Siegel and Driscoll (1995)	0.5	0.5	0	0.5	0.5	0	0.5	0	0	0.5	3
Surya et al. (2017)	1	1	1	0.5	0	0.5	0.5	0	1	1	6.5
Average											5.6
KEY											
	FULLY MET										
	PARTIALLY MET										
	NOT MET										

### CASP Rating Criteria

The findings of the CASP ratings (Table 3) were that there was variability across the final six papers to meet the quality criteria. Ratings ranged from 2 to 9 (with a maximum of 10) and an average rating of 5.6. Many of the studies included were not formal research, rather a qualitative account of programmes and interventions developed in the face of growing recognition of the need for greater staff well-being and support for those who provide care during and after a crisis. The findings of the CASP ratings (Table 3) were that there was variability across the final six papers to meet the quality criteria. Ratings ranged from 2 to 9 (with a maximum of 10) and an average rating of 5. Sub-criteria in the CASP appraisal were not relevant to all papers, as the authors did not set out to meet empirically informed criteria. For example, most studies did not have formal recruitment strategies, although it was implicitly clear why the professionals were being supported and usually the whole crisis team or service was being responded to through the intervention. Further to this, the studies were limited by the crisis context that employed an emergency response that was not planned as formal research and therefore the data was not always systematically collected or analyzed). All papers highlight the imperative to develop support structures and demonstrated the value of the work studied, emphasizing the centrality of the creative elements of the processes used. The need for higher quality studies is clear from the CASP ratings. The selected papers represented a diverse range of settings, carer roles and impactful adverse events, all presenting a rich narrative of the specific responses using descriptive evidence and case vignettes. On this basis we included all six of the papers, referring to the value of the research, and particularly the intervention narrative. It was not possible to draw quantitative data from these studies or for where the CASP rating was low we could not draw on reference to effectiveness, however the primary practice elements were meta-synthesized.

**Table 3:** Adapted TIDieR (Template for Intervention Description and Replication) checklist.

References	Name of Intervention	Why this intervention?	What activities support the intervention delivery?	Who is the intervention being delivered by?	How is it being delivered?	Where is it being delivered?	When is it being delivered, what is the number of sessions and their duration?	Has the intervention been tailored for a particular community?	Were any modifi-cations made during the implementation phase?	How well was the intervention delivered?
Siegel and Driscoll (1995)	Psychodramatic methods for debriefing training	Psychodramatic debriefing methods work well in conjunction with current critical incident training and previously developed relevant psychodrama models	Group training incorporating psychodramatic action techniques such as roleplay, doubling, role reversal and deroling for debriefing.	Police Department's Critical Incident Stress Management Team/ Peer Support by Law Enforcement / Psychodramatist	Face to face training group using roleplay scenarios and follow up assessment session including video	Law enforcement sites, in Mesa, Arizona, USA	One 1.5 to 2 h group session with a follow up session in which the process was assessed and required follow ups were clarified	Structured protocol designed to fit the context. The format of the sessions included some flexibility to meet individual need	Flexibility within sessions to meet individual need. Specific modifications not identified	Assessment administered during the training using a 5-point Likert-type scale for both leaders and team members. The evaluation was used to give feedback to trainees. The intervention was also videoed and critiqued. Assessment forms included but outcomes not included
Ho et al. (2014)	Expressive arts and integrated body-mind-spirit approaches	Disaster management strategy. Schools are often an entry point for offering psychosocial intervention following a disaster. Providing teachers with an intervention protocol to facilitate activities that can alleviate trauma symptoms.	Training in expressive arts and integrated body-mind-spirit approaches	A team comprised of social workers, art therapists, dance/ movement therapists, behavioral health specialists and teachers	Experiential learning. Classroom –based activities	Schools in Hong Kong, Beijing and Wenchuan. Response to an earthquake in Wenchuan, China.	Intensive modules lasting 1-−3 days each with continuous super-vision over 2 years. The training was offered after a year after the 2008 earth-quake	Tailored sensitively to cultural context. Structured protocol allowing some flexibility to support individual group and self-care	Not discussed	Perceived outcomes reported but no formal evaluation of intervention
Huss et al. (2010)	Art Self-regulating tool	1. Historic use of art in healthcare settings with people with high levels of distress. 2. Using art as a self-regulating tool in a war situation. 3. To characterize the stressors of social workers living in a war zone. 4. To teach social workers in crisis situations to identify stress and resilience factors in the artworks and 3)	Introductory lecture on stress reaction as expressed through art. Individual art making on a given theme within a group of peers. A written explanation of their artwork. Group discussion.	Art Therapists/ Social Workers.	Face to face lecture, art making, structured written reflection and structured discussion with a group.	During wartime in the Negev region of Israel during the “Iron Cast Operation” in December 2008 and January 2009.	Single session interven-tions.	Structured protocol designed to fit the context.	Not discussed	Single group case study. Preliminary research with small number of participants. Diverse sources of data including drawings, written explanations of drawings group transcripts and discussion analyzed by participants themselves and three authors creating peer validity. The use of multiple analytical theories increases reliability of findings.
		5. To develop a general self-care model for arts intervention for professionals in these situations.								
Potash et al. (2020)	Art Therapy	Examples of public health best practices for art therapy in pandemics	A range of art and art therapy based interventions including training, virtual studios, creative routines, supervision, psychosocial support groups, art exhibitions, individual and group art and art therapy sessions	Art Therapists	Group and individual Face to face and remote.	Liberia, Ebola epidemic (2014–2015), Hong Kong, SARS (2003), USA, Covid-19 (2020)	Wide range of interven-tions including ongoing and one-off	Numerous interventions were tailored for a range of contexts	Not discussed	Evaluation and outcomes not discussed
Surya et al. (2017)	Art Therapy	To integrate proven mental health strategies to protect the mental health of their workforce and improve staff capacity to provide care for vulnerable populations	A range of art therapy techniques including art therapy as a cognitive behavioral intervention	Techniques and therapies provided by specialist and non-specialist facilitators—for example Art Therapy and Mind—body exercises	Group and individual	International Humanitarian aid organizations responding to complex emergencies in dangerous contexts and extreme environments	Not clearly defined	Generally adapted to the needs of humanitarian aid workers including the use of online and teletherapy methods and confidential self-assessment tools	Not discussed	Evaluation of methods not discussed.
Herbert (2018)	Therapeutic clinical supervision (TCS)	“Creative Arts Therapy Action Research” which engages the organization in a collaborative process in the development of responses to staff care and organizational well-being	Creative Arts Therapy Techniques used in supervision, debriefing and assessment processes developed to support staff-care, wellbeing practice. Group and individual.	Creative Arts Therapists, facilitators and consultants	Face to face group and individual sessions	Cambodia context of post -conflict humanitarian work in Cambodia	Not specified	The action research method involves an intrinsic process of adaptation in response to feedback	Response strategies put in place in relation to emerging individual need during the intervention. For example, a programme of intensive supervision was put in place for a team member who disclosed he was suicidal	Initial outcomes from organizational assessment provided. Perceived outcomes such as a reduction in burnout symptoms mentioned, but no data provided for this.

### Template for Intervention Description and Replication (TIDieR) Checklist

Further to the CASP rating we categorized each intervention according to the TIDieR checklist (Hoffmann et al., 2014) to determine the scope for replication of each intervention description and to ensure that the papers fulfilled the required inclusion criteria. Whilst the descriptions of the interventions within the papers met more of the checklist criteria than the CASP rating, the lack of evaluation of treatment fidelity was apparent across all studies as were clear descriptions of any modifications that were introduced during the intervention implementation phase (See Table 3).

## Thematic Analysis

### Arts-Based Crisis Intervention Change Process Elements

**Figure 3:**
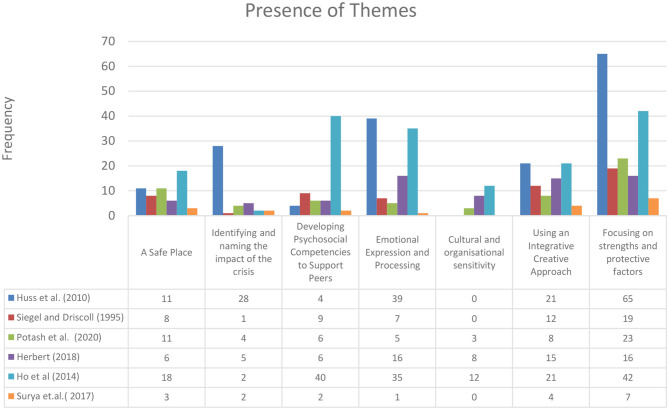
Presence of arts-based crisis intervention change process elements themes.

The interventions employed during or after the crisis were thematised according to the author's description of the therapeutic process and what was considered as being central to meeting the aims or goals of the intervention (See also Table 3). There were 573 excerpts coded with 256 codes which were applied 713 times. This produced 17 superordinate codes and 7 themes (Table 4). The themes carried across all the selected papers with logical rationales except for cultural and organizational sensitivity, which was coded in 3/6 papers (See also Figure 3).

**Table 4:** Superordinate codes and themes for change process elements of the arts-based crisis interventions.

Superordinate Code	Theme
Using a directive/structured approach	A Safe Place
Safely sharing experiences/developing trust	
Enabling self-care/supportive	Focusing on strengths and protective factors
Identifying resources	
Problem solving /recovery/	
Supportive	
Recovery approach	
Cognitive focus/ reframing	
Developing carer psychosocial competencies to support others	Enabling and supporting participants to provide care and support
Enabling expression and processing	Processing the emotional response to the impact of the crisis
Psychoeducation	Identifying and naming the impact of the crisis
Naming, assessing and monitoring impact of crisis	
Creative/integrative approach	Using an integrative creative approach
Psychophysiological engagement	
Collaboration	
Systemic approach	Cultural and organizational sensitivity
Culturally/ prejudice sensitive	

### Theme 1. A Safe Place

A safe place comprising two themes that offered a culturally nuanced approach and highlighted the importance of feeling safe during or after a crisis. This theme had 47 applications across all the selected papers. This also meant not re-traumatizing participants and enabling sufficient structure and direction to feel enabled to fulfill the tasks safely.

#### Theme 1a. Finding Safety

Facilitators of interventions considered that the development of a place that felt safe during or after a crisis was a primary task. The method for how this was achieved depended on the context, for example in a teaching context following a disaster in China (Ho et al., 2014), the cultural idea of a safe place was laden with patriarchal values and new cultural values about what initiated a safe context were required to be introduced in a way that would not overly challenge assumptions about caring and teaching roles. For example, the facilitators used an exercise of clipping flowers to make artwork as a non-verbal expression of respect for the deceased. An intervention of experiential learning was introduced that was structured according to the emotional needs of the teachers and their students. In Cambodia, a key issue was addressing shame and humiliation for how some members of staff felt about how they were coping, which sometimes included being violent toward others. In order to do this, the primary task of the crisis interventions of finding a safe place was pertinent to supporting their role. As Surya et al. (2017, p. 188) state, “The mental and psychological well-being of health professionals is imperative to their ability to function effectively particularly when exposed to extreme environments.” Again, Potash et al. (2020, p. 1) describes art therapy in pandemics, stating the importance of “engendering a sense of control in a safe place.”

#### Theme 1b. A Structured and Directive Approach

In all contexts, experiential learning was a key element of the crisis intervention as well clear directives, duration and learning outcomes. Herbert (2018, p. 230) describes the structure as ritual and ceremony in a Cambodian context and again focus on the cultural context that felt safe and preventative “…without leaving a person retriggered or overwhelmed by their experience.” (Herbert, 2018, p. 230). Similarly, Siegel and Driscoll (1995) describe a seven step approach to processing critical incidents for emergency personnel. The authors illustrated sensitivity to the potential harm caused by an unstructured exploratory space and one of the aims to be to “gain emotional distance from the event” (Siegel and Driscoll, 1995). This is an example echoed in the other papers of providing a clear structure, aims and goals for the intervention.

### Theme 2. Focusing on Strengths and Protective Factors

Strengths and protective factors were the largest theme with 107 code applications and significant overlap with other themes (See **Figure 5**). This theme comprising two subthemes, that included the capacity to mobilize personal, social, and organizational resources toward problem solving and ensuring self-care and the care of others.

#### Theme 2a. Self-Care

Surya et al. (2017) state that the importance of identifying and developing strengths and protective factors during and after crisis situations is of uppermost importance. Given that organizational systems and communication may not be as effective as need be during a crisis, many of the resources available that will reduce the impact of stressors need to be realized in a way that supports the individual, team and organization (Herbert, 2018). This includes a capacity to use the arts to support a caring environment. In fact, the dramatherapist facilitating a drama process for emergency staff, stated that they based the therapeutic action on “peer support” rather than psychotherapy, preventing “cumulative stress.” Surya et al. (2017) describe a holistic approach during or following a crisis to develop a caring environment to enable healthcare workers to feel “…it is really OK to break for a cup of tea” when the stress became too much. They argue that it is common for healthcare workers not to feel that it's OK to be well when people around them are suffering. Huss et al. (2010, p. 206) also stress the importance of self-care as a strength of the participants when they describe encouraging social workers in a war zone to “use art as a reflective and transformative tool” that can be “conceptualized as a potent method of self-care and self-nurturance.”

#### Theme 2b. Identifying Resources and Problem Solving

As part of a recovery process, identifying resources and problem solving were considered as central to an arts-based approach. As Ho et al. (2014, p. 276) describes, “The recovery from the trauma lies partly in regaining a sense of self through identifying resources and strengths, reorganizing one's world view and mobilizing coping as a foundation for future growth.” This means that authors described the use of arts and particularly art as an object that could be both an expression, but also represent the ways in which the person could cope, either by using the art object to reflect internal resource for coping, or to demonstrate how the situation could be looked at differently and offer ways of adapting, reframing or solving a problem.

As Huss et al. (2010, p. 204) state, “…the social worker was encouraged to identify sources of coping and resilience.” For example, one social worker used an image she made to focus on during sirens that alerted the community to air strikes. Huss et al. also look at how expressions in the artwork are altered to reflect potential ways in which real-world problems can be solved. Ho et al. (2014, p. 280) describes a group of teachers regrouping at the end of their work together, holding hands and rhythmically circling reassembled art “to document their strengths and enhance their community connections.”

Art therapy for healthcare workers was also recommended as one of several therapeutic crisis interventions that enhanced “creativity, flexibility, problem solving, and coping skills” (Surya et al., 2017, p. 192). These activities appeared to support the mobilization of internal resources toward adapting to the crisis context, whether this involves for example “reviewing roles” using psychodrama after a critical incident (Siegel and Driscoll, 1995), or reframing the emotional expression in the art work following a crisis to find “an alternative more adaptive response” (Huss et al., 2010, p. 207).

### Theme 3. Developing Psychosocial Competencies to Support Peers

As mentioned previously, a primary aim of arts-based crisis interventions during and after crises is to enable carers to successfully fulfill their responsibilities, which was referred to 42 times across six papers. Whilst much of the work is conducted through group workshops and educational programmes, the arts-based practitioner often provided support in a supervisory role to enable the application of fundamental principles of recovery. For example, in post-war Cambodia, much of the arts-based practice happened through a supervisory role where skills could be passed on. Herbert (2018) describes the impact of principles of creative supervision practice, “Her approach was one that modeled non-judgmental therapeutic support and presence. Within the structure created by the team leader, the member of the team disclosed to her that he was suicidal. In response to this, the team leader put in place a response strategy and supervision support.”

It was apparent that in all examples of the use of arts-based crisis interventions, this wasn't only developing existing skills and knowledge, but providing new learning that could be internalized and utilized for others. In some contexts, such as with the Chinese teachers, traditionally their role wasn't to care for traumatized children; the acquired skills required a change in approach, role and understanding. This meant that teachers not only participated in arts-based experiential workshops, but that they ran similar activities for the children, utilizing the emotional understanding that they had gained through processing their own traumas, “…when mastered, the activities can be shared and taught to others” (Ho et al., 2014, p. 281). In other contexts, caring for others was a more explicit part of their defined role, for example, in the law enforcement context, debriefing and working through trauma was led by peers that are supported and facilitated through psychodrama as part of the workshops and that was otherwise part of the culture of the unit (Siegel and Driscoll, 1995). However, the facilitation helped them to develop a structured approach to caring for their peers that would otherwise not have happened in such a systematic and focused way.

### Theme 4. Emotional Expression and Processing

Given that crises are defined by the emotional and interactional language within the social context, it is unsurprising that understanding emotional expression, communication and processing this experience was the second most cited change process element, being referred to 79 times across six papers. There are several ways in which the review revealed the expression and processing of emotional experiences through arts in the selected papers. Non-verbal expression was described as enabling the communication and processing of emotions through symbolisation and verbalisation of experience (Huss et al., 2010, p. 207). Further to this, the emotional connection to others was enhanced and developed, providing a resource and shared experience of the trauma (Ho et al., 2014). Herbert also (2018, p. 230) states that the expression of emotions through the artwork as a way of symbolizing, “…enabled contained expression and extraction of emotions from the body and heart onto paper.” In China, the teachers used the arts to understand the “emotions and spiritual concerns behind the presenting behaviours,” meaning that they could reflect on their own experience more fully, but also empathize with their students more effectively. Normalization of emotional reactions was also a frequent theme that appeared across all the papers, for example, that “… outrage, horror, shock, sadness, or vulnerability is normal…” (Herbert, 2018). The review described the application of arts-based interventions in organizations and cultures where expression of emotional reactions to trauma was highly stigmatized. Therefore, the processes that facilitated emotional expression often cultivated cultural activities that felt normal and were not explicitly described as therapy in any of the contexts. Instead, peer facilitation (Siegel and Driscoll, 1995), supervision (Herbert, 2018), arts activities (Ho et al., 2014), lectures (Huss et al., 2010), and psychoeducation (Surya et al., 2017) were terms used to describe elements of the arts-based approach, rather than “arts therapies” or “psychotherapy,” even where a primary outcome was therapeutic change. In this sense, the arts responded to a cultural context to enable the safe expression of emotions with an informed understanding of how to support others.

### Theme 5. Identifying and Naming the Impact of the Crisis

This theme occurred 37 times across six papers. Linking emotions and the social behavior to the context of the crisis was described in all the papers reviewed. This means that how events, such as loss of family and friends, disruption to care and social systems, social disorganization, media coverage and prolonged exposure to people's suffering were all identified as affecting the role of the carer. In all papers in this review, they give participants the resources through an educational component to identify how the crisis may have affected them. For example, Huss et al. (2010) provides arts-based psychoeducation for social workers, focusing on trauma and the impact of working in war zones. Potash et al. (2020) also describe the wider role of arts in educating populations about the impact of pandemics, to counter the impact of social media and news that might provoke fear and anxiety. In Cambodia (Herbert, 2018), there was a description of a thorough collaborative analysis of the needs of the individual, team and organization following the crisis. “[Through creative methods, this]…enables an assessment of the overall culture for care for staff in the organization, assessment of the staff care policy, of existing levels of stress and vicarious trauma, individual, team, and organizational coping strategies and support systems.” (Herbert, 2018, p. 231). In China there was considerable bereavement across the community, and the impact of this was considered before providing a crisis intervention. With a large community or organization, this happened prior to the work, however identifying the impact also featured as a structured part of the work, for example, for emergency personnel assessment of the impact was considered in the “reaction phase” of the crisis intervention and particularly to identify causal factors, symptoms and the sources of their stress (Siegel and Driscoll, 1995). At the beginning of the crisis intervention, “stress” was commonly used to summarize the impact of the crisis, however, the quality of the felt experience, including emotional and mental health experiences that may result from the crisis were revealed in more nuanced and explicit ways during the crisis intervention. Huss et al. (2010) describes using the image-making process, for example noticing disjointed images, fragmentation and jagged lines to help identify indicators of high levels of stress. Recognizing the link between the sources of stress and the expression of the stress is also supported through psychoeducation, where the participants are informed about what can happen during a crisis and its potential impact on health and well-being and what strategies are required to offer sustainable change (Ho et al., 2014).

### Theme 6. Using an Integrative Creative Approach

This theme occurred 51 times across all six papers. Whilst there was a clear structure of crisis intervention described in each paper, the flexibility of adaptation to needs, the use of creative mediums and the versatility of the tools were integrated in collaboration with the participants. The aims were to provide a method that was acceptable, accessible and produced meaningful, if not transformative change “that enables participants to go beyond words, express what is seen and unseen, reflect deeply and so bring informed learning and recommendations for what is deeply needed in the field” (Herbert, 2018, p. 231). Part of the rationale was to utilize arts that enable physiological and psychological change through engaging in “different levels of intervention” (Huss et al., 2010, p. 202) and provide a “holistic approach” to care (Ho et al., 2014). The rationale of using a holistic approach is that stress or trauma remains unverbalised and often body bound, requiring ways to enact and express experience through non-verbal means. They base a second dimension to the versatility of using arts on the cultural acceptability, where standardized and often individualized forms of therapy are not accepted or are taboo. Drawing, painting (Huss et al., 2010; Herbert, 2018), using cultural artifacts (Ho et al., 2014), rituals and ceremonies (Herbert, 2018) are adopted as vehicles of social expression within a learning structure. Therefore, an integrative approach includes crisis interventions that provide a combination of different arts modalities (Ho et al., 2014; Herbert, 2018) physiological and psychological engagement (Huss et al., 2010; Herbert, 2018) as well as cultural and community-based practices (Ho et al., 2014; Herbert, 2018). Further to this an integrative approach is also defined by the collaborative nature of the intervention Huss et al., 2010 including seeing the learner as teacher (Ho et al., 2014), and as “action researcher” of their own work (Herbert, 2018) and a participant that can also provide similar activities for others (Ho et al., 2014; Herbert, 2018). In summary, the crisis arts-based interventions utilize creative and versatile methods to address constantly changing complex personal, organizational, and social needs.

### Theme 7. Cultural and Organizational Sensitivity

We identified cultural and organizational sensitivity 12 times across three papers. As a crisis is defined by the social interactions in response to an unexpected event that has a disruptive impact on social and care systems, the cultural context is important. In some contexts, arts were considered as being more culturally acceptable than verbal interventions, where there is a cultural heritage of healing arts, such as singing, embroidery, brush work and body movement (Ho et al., 2014). As (Herbert, 2018, p. 226) puts it, “The arts naturally contextualize into individual and collective culture and provide an inherently natural medium within which to explore complex systemic issues.” The other side of this is that there are often care workers who feel that they exist outside of the cultures of the people that they are caring for and that existing mental health or care services are not meant for them (Potash et al., 2020) and their usual informal support systems are no longer available due to the crisis. As described previously, the review suggested that stigmatization relating to mental health services and psychological treatments is also an issue for some cultures (Ho et al., 2014). Within these contexts, the therapeutic use of arts can offer an alternative approach to verbal interventions (Potash et al., 2020).

### Presence of Themes for Arts-Based Crisis Intervention Change Process Elements

**Figure 4:**
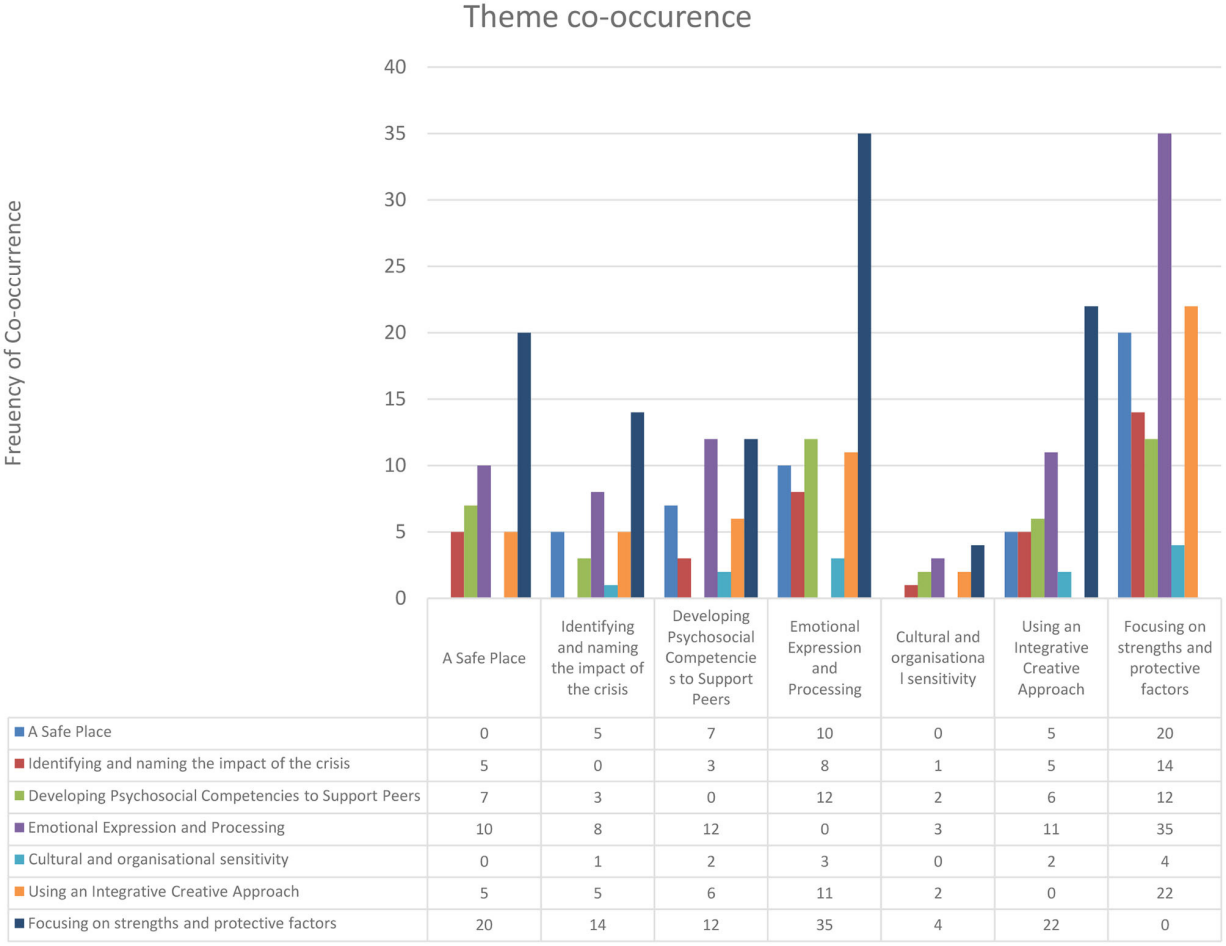
Arts-based crisis intervention change process elements theme co-occurrence.

Out of the seven change process elements, we identified six of them that were present across the papers, with a high presence of focusing on strengths and protective factors as well as emotional expression and processing. The only practice element that was not present in all papers was cultural and organizational sensitivity, which appeared in three papers (See Figure 4).

### Theme Co-occurrence

**Figure 5:**
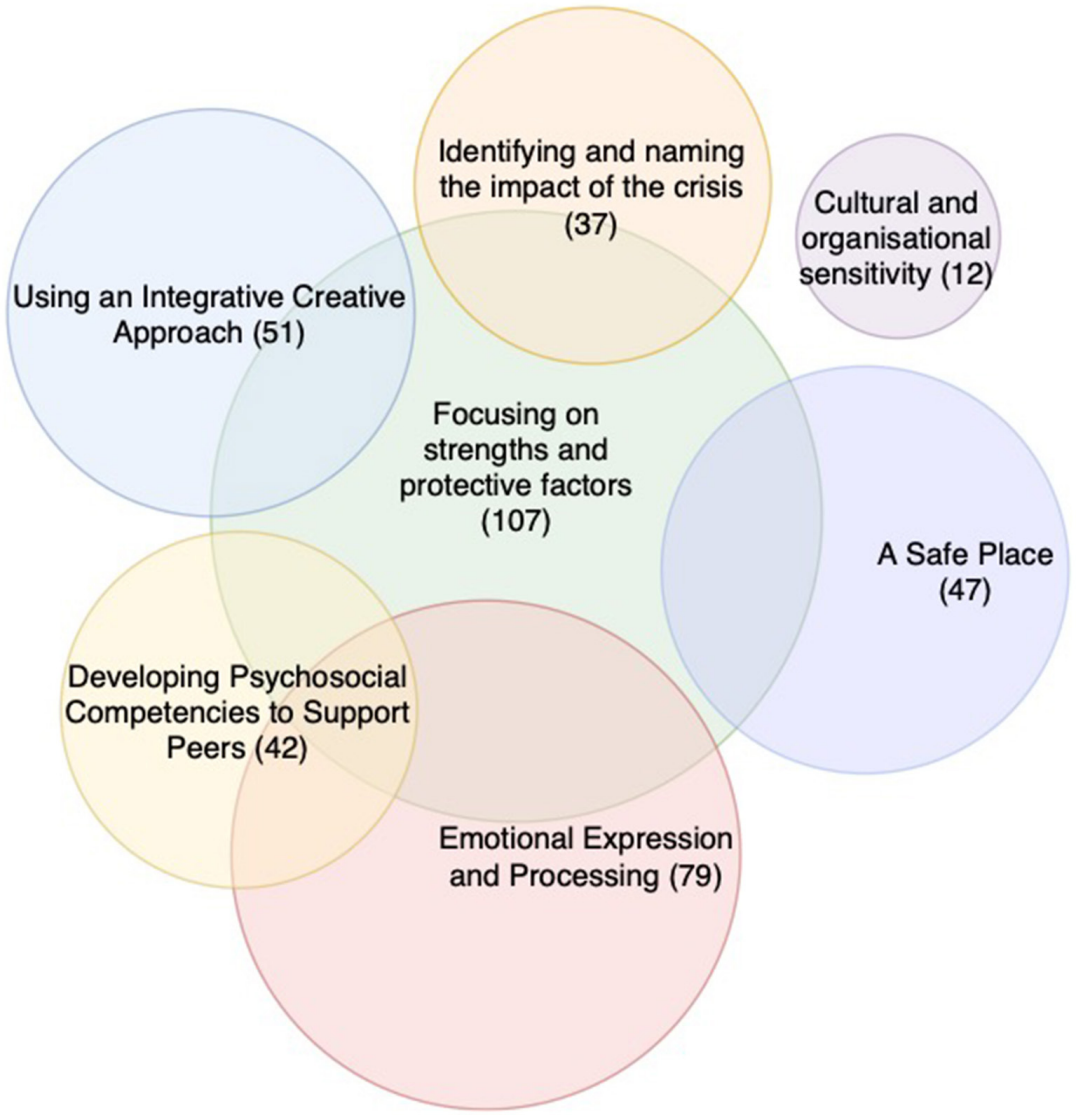
Theme co-occurrence Venn diagram (<25% co-occurrence).

We applied the themes 545 times across the papers, with relatively low co-occurrence suggesting that the themes are clearly delineated. The highest prevalence of co-occurrence was between focusing on strengths and protective factors and emotional expression and processing, suggesting that the nature of strengths and protective factors were frequently identified with an emotional experience. It is also notable that cultural and organizational sensitivity was the least coded and had the least co-occurrence across the papers. Where there was over 25% co-occurrence, we have illustrated these in Figure 5. The findings showed that focusing on strengths and protective factors co-occurred with all other themes.

## Intermediate and Long-Term Perceived Benefits

The long-term perceived benefits in all papers (See Table 5) described personal and community post-trauma growth, as a process of recovery that involved personal insight and change. The review also described a process of adaptation, often challenging personal and social norms through acceptable use of the arts, enabling a greater level of care, emotional openness and communication amongst care professionals. Developing skills to process psychophysiological reactions to trauma was perceived to impact on the prevention of mental health issues and, where there were already mental health issues, reduced stress or trauma reactions.

**Table 5:** Superordinate codes and themes for perceived intermediate and long-term benefits.

Superordinate Codes	Themes
Change of self-perception	
Post-trauma growth	
Adapting to change	
Recovery	
Developing resilience	Adapting growing and recovering
Community development	Using the community as a healing resource
Reducing / preventing symptoms of stress/ trauma	Reducing or preventing symptoms of stress or trauma reactions
Enabling expression and processing	Psychophysiological Homeostasis
Affect regulation	
Creative / integrative approach	
Well-being	

Symptom change is important to consider regarding sustainable change and impact. The selected papers focus on sustainability of the intervention based on group work, community involvement and the sharing of skills and understanding within the workforce to prevent or reduce mental health symptoms. Further to this, the review findings suggest that the approaches are rarely focused on the individual and usually work through group and community structures.

### Theme 1. Adapting, Growing, and Recovering

Engaging in an arts-based crisis intervention required adapting to new ways of working, contexts and ways of relating to peers and those that were being supported or cared for. This sometimes meant that changes to the person's role and self-perception were required that would benefit the community in the longer term. For example, in China teachers are traditionally considered within a paternalistic model of hierarchy, and the focus of emotions and trauma would not usually be considered within the role. However, the intermediate changes included adaptations to the teachers' role, providing innovative and creative ways of engaging with children who had experienced traumas, and in the long-term the teachers enabled a change to the culture of education. Similarly, where social workers rarely provided self-care as a part of practice as usual, the intermediate impact was on receiving the skills to be creatively self-reflective, that included the use of arts to facilitate their recovery independently of a facilitator. The impact within the team, offered a change to the culture to include self-care in social work in a war situation (Huss et al., 2010) and that sharing painful emotional experience can become a normalized part of the team culture and community growth (Huss et al., 2010; Ho et al., 2014; Herbert, 2018).

### Theme 2. Psychophysiological Homeostasis

Linked with growing and recovering through adaptation was the theme of how trauma was being processed to positively impact on key areas requiring a condition of psychophysiological homeostasis. This review suggested that the capacity to process trauma at a psychophysiological level had the result of enabling people to resume their work when they had otherwise been physically and emotionally debilitated by traumatic responses (Herbert, 2018). Within a war context, (Ho et al., 2014, p202) describe the use of drawing as a “… way for social workers to engage in self-reflection on their signs of stress and as a way to bolster resilience and coping to counter intense psychophysiological reactions to such a crisis situation as war.” Similarly, the intermediate benefits of the use of arts as a way of expressing and reflecting on experience, preventing destabilization is described by Surya et al. (2017) as “…enhancing creativity, flexibility, problem solving and coping skills….” Underlying the sustainability of the intervention was the mobilization of deep reflection and learning (Herbert, 2018).

### Theme 3. Using the Community as a Healing Resource

The value of developing community cohesion and growth was often a larger aim of the intervention, beyond the team and organization. This was to do with the scale of the impact being on a cultural and social level beyond the immediate people being attended to. This meant that the arts were used to generate cohesion through social media posts and celebrating community achievements through the arts, where a primary aim was often to develop “solidarity” (Ho et al., 2014; Potash et al., 2020). This was partly to do with the use of arts being employed in some collectivist countries (Ho et al., 2014), that focused on the wider social context rather than the individual, however community development was also considered as part of an ongoing process of “enhancing community connections,” and “rejoining… and… rebuilding” (Ho et al., 2014). For example, part of a process of mourning for teachers was of considering the legacy of those that had died and considering the wishes of the deceased as a way of thinking about the future of the community (Ho et al., 2014). This linked with how care professionals felt that they could make a wider contribution to the community than had previously been considered within their role (Surya et al., 2017).

### Theme 4. Reducing or Preventing Symptoms of Stress or Trauma

The review showed that there were a range of symptoms that were perceived to be reduced through engaging in experiential arts-based programmes. The most frequently mentioned issues that were impacted on were stress and trauma reactions, noted as being as effective as cognitive behavioral therapy (Surya et al., 2017). Further to this, a key aim was to enable communities and teams to support each other to prevent the reoccurrence of stress reactions “by helping their peers to understand the physical, cognitive, emotional and behavioral responses to traumatic events” (Siegel and Driscoll, 1995). Prevention is a recurrent theme where the recognition and assessment of mental health issues amongst carers is crucial and the prevention of “…long-term emotional and physical illness…” (Siegel and Driscoll, 1995) or the amelioration of “deeply set psychological traumas…” (Herbert, 2018).

## Discussion

### Main Findings

This review provided a systematic thematic analysis of the elements that make up arts-based interventions of practice for communities in crisis. We have thematised primary change process elements and perceived benefits through examining papers published from 1995 to 2020. The findings highlighted the importance of a structured collective focus that can utilize the arts to process complex emotional states and to support skills and knowledge development for professionals. Addressing the first question in our review, the main findings revealed that there are shared change process elements that use arts to facilitate change where communities have been deeply affected by the loss of people and support systems. The publications to date report that the importance of establishing a sense of safety, often using a structured approach, for example within a psychoeducation intervention, is a premise of developing trust and exploring emotional experiences. Many of the challenges within a crisis are to do with the struggles to adapt given limited resources and damaged infrastructures, which meant that a collaborative approach to problem solving was an integral part of the work. The facilitators were also explicit that the arts were being used in the service of facilitating communication and developing skills that would enable participants to be caring for colleagues within the scope of their professional roles. The assessment and monitoring of the impact of the crisis was a key feature of the interventions of practice, as was the careful facilitation of culturally sensitive emotional expression, processing and sense making through the arts. In relation to our second primary aim, all of the papers reviewed suggested that there was an impact on the symptoms of trauma and stress and that this could be sustained and prepare the workforce for future social crises. Whilst we found limited data on the effectiveness of these interventions, case studies and reports suggest that they were well-received and reports of positive change confirm the hypotheses that the arts can successfully facilitate support, coping, and professional role adaptation during and after a crisis event.

The role of arts during and after crises for communities is often about helping people to reflect on events, express emotions and narratives, develop community cohesion through participation and offer insights into alternative possibilities and solutions (Campbell, 2005; Bal, 2020; Marandet et al., 2020; Richeson et al., 2020). This review demonstrated that some primary elements that enable recovery and growth following a crisis can be responded to and may be mobilized through the use of arts. In a paper published after we conducted this review, the president of the Royal College of Nursing, Professor Anne Rafferty, stated that “the arts, ‘.help us face the huge uncertainties we are confronting.' The arts, she says, are a route to building ‘a sense of resilience, a sense of solidarity, a sense of strength”' (Mermikides, 2020). We suggest that according to cultural values the arts can be acceptable forms of intervention and given the high prevalence of arts-based activity during and following crises that the application of arts-based interventions should be further researched for the needs of caring professionals during or following a crisis.

### Methodological Limitations of the Review

The limitations of this review primarily relate to the rigor of the data provided by the reviewed papers. Most papers employed a heuristic approach to investigation and description of practice grounded in a phenomenological theoretical position. The cultural and professional contexts were very different, and whilst similar themes emerged, the cultural nuances would need further data to confirm the appropriateness of the interventions to the context. The intention to describe elements of the change process is challenging given the overlap of immediate outcome and therapeutic action being very similar, that is, the intervention was commonly described as developing skills which could be used by the participant to help others therefore the input of the practice model could not be differentiated from the output. Lastly, whilst perceived benefits were reported as being positive, to date there are no effectiveness studies to verify this.

### Recommendations for Assessing and Evaluating Arts-Based Interventions for Professionals in Caring Roles During and After Crisis

The impact of social crises can take a long time to recover from and often the psychological effects are not felt until months after the event. Individualized psychological interventions are not specifically designed for social trauma and are not known to be acceptable within collectivist cultures (Boreham, 2004; Furedi, 2004; Bauman, 2008; Kira, 2010). Kira (2010, p. 137) suggests that “This understanding entails revising our culturally limited and single personal identity–based interventions to help clients who belong to different cultures or minority victims of culture- and social-made traumas as well as those who are victims of cumulative traumas.” One finding of the review is that enabling novel ways of working for care professionals requires interventions that support the collective health and provide skills and knowledge that can facilitate adaptive changes to the social environment. Second to this, the impact of crises is commonly greatest in urban environments, affecting a wide range of cultures. Therefore, further research should be conducted to determine the acceptability of the use of arts within a range of different health contexts.

This review describes the role of arts to support professionals within their working environments across a range of contexts during or following a crisis. Whilst these studies indicate positive changes for participants, future studies should investigate the acceptability and perceived benefits of arts-based interventions in the event of a crisis, for example, a future pandemic, natural disaster or acute social conflict. Early findings suggest that crises often exacerbate existing issues regarding communication and social interaction and therefore arts-based interventions should also be researched for their effectiveness to support care workers in acute care contexts to build resilience and prevention of mental health issues and supporting communication, culture change and professional cohesion. An equally important area for the further research of arts-based interventions in this context is to test the change process elements identified by research methods and underpinned by cohesive theoretical modeling to investigate the scope and range of creative arts-based interventions.

Theoretical modeling of arts-based practice should be further developed using the themes defined in this paper for specific populations, especially in situations where a crisis has a collective impact. At present there are limited tools available for measuring changes to the social context, however social identity theory could provide scope for measuring the collective self-esteem (Luhtanen and Crocker, 1992), group identity (Brown et al., 1986; Karasawa, 1991; Henry et al., 1999; Jackson and Smith, 1999a), competitive intergroup comparisons (Ouwerkerk et al., 2000), social identity and interdependency (Jackson and Smith, 1999b), and perceived homogeneity (Pickett and Brewer, 2001). Cultural assessment (A Simple Organizational Culture Assessment Questionnaire, 2019) and changes to social relationships (Aguilar-Raab et al., 2015) can also be used in a range of environments. We recommend that the ideal cultural and social functioning is decided by the group and compared with pre and post measurements. Further to this, collective change could be compared with individualized data regarding compassion fatigue (Campbell, 2013), burnout (Morgantini et al., 2020), PTSD (Seligowski et al., 2015), depression and anxiety. Given the complexity of an arts-based intervention, that, according to this review, was often adapted in collaboration with the participants (Ho et al., 2014; Herbert, 2018; Potash et al., 2020) the authors would suggest using a research method that is pragmatic and context sensitive. The research design would need to be sensitive to ethical considerations of providing interventions within care contexts. For example, a step-wedge cluster-randomized community-based trial (De Allegri et al., 2008; Joag et al., 2019) may be a good design to provide comparative data about individual and social change. Whilst researchers have identified the problems of context, culture and accessibility, intervention development and research methodologies have often remained individualized (for example, see Selamu et al., 2017; Joag et al., 2019). We return to the definition of a crisis as one of disrupted social communication and interactions. We believe that if the social premise of sustainable change is to be effected, further research should include social interaction and communication in relation to professional role and health as measurables within the study. There are a range of studies that investigate effective communication for care professionals in terms of the competence (See Stern, 2006; O'Daniel and Rosenstein, 2008; Ang et al., 2013) and the organization of care, including communication (Seys et al., 2013). However, further research is required to address effective and caring communication as one of the main mediators of change for this population following a crisis. From this review the thematic meta-synthesis indicates that therapeutic targets for the care worker population should include a change process that focuses on a combination of recovery principles, community development, psychological and physiological regulation, community well-being and mental health and physical illness prevention and reduction, particularly in the areas of stress, traumatic reactions and depression. Further to this, this review suggests that arts can be used to increase cultural sensitivity and treatment accessibility. We consider these areas of the intervention a priority for future research given the potential for scalability as suggested by these studies which were delivered within a diverse range of international contexts. The professional role is also an important moderator of change and should be taken into account in future effectiveness research studies given the importance to participants that the intervention supports professional knowledge and conduct.

## Conclusions

This review suggests that whilst there are few publications to date, there is evidence to indicate that not only can arts be used to support care professionals but that there are shared perceived benefits. There are no quantitative studies to date that investigate the effectiveness of arts-based interventions for healthcare workers during and after crises and the quality of the existing studies suggests that this is an area that requires more robust studies to be conducted. Highlighted in this review were the findings that a safe place, focusing on strengths and protective factors, developing psychosocial competencies to support peers, emotional expression and processing, identifying and naming the impact of the crisis, using an integrative creative approach, and cultural and organizational sensitivity were an integral part of the theoretical modeling used to impact on perceived outcomes. The specific change process elements that the reviewers identified appeared to be shared across all identified studies, with the exception of cultural and organizational sensitivity, suggesting that these elements may be integral to the required change. Further to this, the arts-based interventions identified in this study appeared to be low cost and scalable interventions for reducing mental suffering and promoting well-being during and after crisis in a wide range of settings.

## Data Availability Statement

The data that support the findings of this study are openly available at https://figshare.com/articles/dataset/_/13187138.

## Author Contributions

DH-F coordinated the data collection, study design, led the manuscript development process, carried-out the analysis of data, and development and writing of the drafts and manuscripts. MT coded papers supported thematic analysis and made contributions to the paper. JK coded papers, supported thematic analysis, coordinated the TIDieR data, and assisted with data collation. CG independently rated the papers using CASP and made contributions to the paper. VK oversaw the findings, further research, recommendations, and made contributions to the paper. All authors contributed to the article and approved the submitted version.

## Conflict of Interest

The authors declare that the research was conducted in the absence of any commercial or financial relationships that could be construed as a potential conflict of interest.
